# PP102 Sustaining Ghana’s National Health Insurance Scheme: Leveraging The Role Of Priority-Setting Tools In Benefit Package Reviews

**DOI:** 10.1017/S0266462324002642

**Published:** 2025-01-07

**Authors:** Ruby Aileen Mensah Annan, Lieke Fleur Heupink, Richmond Owusu, Katrine Frønsdal, Genevieve Aryeetey, Lumbwe Chola, Patricia Akweongo

## Abstract

**Introduction:**

Ensuring the National Health Insurance Scheme’s (NHIS) sustainability is essential to achieve Universal Health Coverage in Ghana. However, numerous addition requests to the Benefit Package (BP) places the scheme in precarious situations, necessitating evidence-based prioritization. Our objective is to conduct a situational analysis on the role of health technology assessment and priority-setting within the National Health Insurance Authority (NHIA).

**Methods:**

Qualitative methods were used to assess the current NHIS BP review process. Desk reviews of policy documents and key informant interviews to establish the mandate of the NHIA in BP reviews were conducted. Finally, a mapping of the alignment of evidence from HTAs conducted by the Ministry of Health (MOH) with recent BP review and inclusion decisions of the NHIA was carried out.

**Results:**

Findings validate the legal mandate of the NHIA to carry out BP reviews with processes spelled out in operating guidelines. Actuarial analysis determines the scheme’s capability to fund additional service, but no explicit priority-setting criteria are used. This leaves the NHIA open to external pressures for inclusion and presents risks to the financial sustainability of the scheme. An HTA on antihypertensive treatments in 2017 influenced reimbursement pathways of the NHIA. Similarly, the cost-effectiveness of extending existing medications on the NHIS Medicines List to cover Burkitt lymphoma corroborated the decision of the NHIA to cover childhood cancer in 2021.

**Conclusions:**

The NHIA uses actuarial evidence in BP inclusion decisions. It, however, lacks an explicit priority-setting criteria to guide decisions on coverage. HTA evidence has been leveraged for sustainable coverage decisions. The development of an explicit priority-setting framework for reviews can guide inclusion decisions as well as clarify how the NHIA aligns with the HTA generation body.

